# Reliability Design and Electro-Thermal-Optical Simulation of Bridge-Style Infrared Thermal Emitters

**DOI:** 10.3390/mi7090166

**Published:** 2016-09-13

**Authors:** Peng Zhou, Ranbin Chen, Na Wang, Haisheng San, Xuyuan Chen

**Affiliations:** 1 Pen-Tung Sah Institute of Micro-Nano Science and Technology, Xiamen University, Xiamen 361005, China; zhoup@stu.xmu.edu.cn (P.Z.); chenranbin@126.com (R.C.); pepperwn@foxmail.com (N.W.); 2 Department of Micro and Nano Systems Technology, Buskerud and Vestfold University College, Horten 3184, Norway; xuyuan.chen@hbv.no

**Keywords:** thermal emitter, infrared emission, finite element method, micro-electromechanical systems

## Abstract

Designs and simulations of silicon-based micro-electromechanical systems (MEMS) infrared (IR) thermal emitters for gas sensing application are presented. The IR thermal emitter is designed as a bridge-style hotplate (BSH) structure suspended on a silicon frame for realizing a good thermal isolation between hotplate and frame. For investigating the reliability of BSH structure, three kinds of fillet structures were designed in the contact corner between hotplate and frame. A 3-dimensional finite element method (3D-FEM) is used to investigate the electro-thermal, thermal-mechanical, and thermal-optical properties of BSH IR emitter using software COMSOL^TM^ (COMSOL 4.3b, COMSOL Inc., Stockholm, Sweden). The simulation results show that the BSH with oval fillet has the lowest stress distribution and smoothest flows of stress streamlines, while the BSH with square fillet has the highest temperature and stress distribution. The thermal-optical and thermal-response simulations further indicate that the BSH with oval fillet is the optimal design for a reliable IR thermal emitter in spite of having slight inadequacies in emission intensity and modulation bandwidth in comparison with other two structures.

## 1. Introduction

In the last years, non-dispersive infrared (NDIR) gas analysis systems have been widely used in air quality monitoring, gas leak detection, fire detection, and disease examination. Infrared (IR) light sources, which are key component in the IR gas analysis systems [1], are required to have a broad emission spectrum covering wavelength range from 1.5 to 20 μm. Traditional IR sources include IR lasers, IR light-emitting diodes (LEDs), and thermal emitters. The IR lasers are of high cost, and the IR LEDs are of low emission intensity in mid-IR range, thus the two kinds of emitters are not the best choice for NDIR gas analysis systems [2,3]. Thermal emitters such as light filaments can meet the requirements of low cost and high emission intensity, but it is difficult to achieve a high modulation frequency that is indispensable for harmonic detection used in IR analysis systems.

Modern micro-electromechanical systems (MEMS) technology can be used to produce the high thermal-mass of microstructures [4,5]. Since the late 1980s, micro-heaters based on closed hotplate structure have been investigated as substrates for metal oxide gas sensors. In recent years, this potential has been used to produce micro-machined thermal emitters for NDIR gas analysis systems [6,7,8,9]. Up to now, the commercial MEMS-based thermal emitters, such as thermal emitters from Intex (Intex Inc., Tucson, AZ, USA) and Axetris (Axetris AG, Kaegiswil, Switzerland), have been developed using closed hotplate structure with operation temperature in excess of about 600–700 °C and modulation frequency in excess of about 15 Hz [10,11]. However, these thermal emitters still need to further improve performance in decreasing power consumption and increasing the electrical-to-optical conversion efficiency and device reliability. For these reasons, the MEMS-based thermal emitters based on suspended hotplate were also investigated [6,8,9]. These suspended hotplates are of lower power consumption, faster thermal response, and higher emission intensity than the closed hotplate using same dimensions, but the fragile support structures easily suffer from cracks and breaks due to the thermo-mechanical stress and fatigue induced by the electric pulse loading [12]. In our previous work [13], a bridge-hotplate structure IR emitter based on MEMS technique was designed and fabricated, which demonstrated higher photoelectric performances than the closed-hotplate structure IR emitters. However, there are still reliability problems during thermal-cyclic operation of the device. For example, some devices failed to work due to the damage of hotplate induced by the high thermal-mechanical stress in local structures during the operation of electrical modulation. Therefore, there need to design an optimal hotplate structure with improved thermal performance and reliability.

In this paper, we designed a bridge-style IR thermal emitter with optimal fillet structure. Three-dimensional finite element method (3D-FEM) is used to simulate the performances of thermal emitters by using software COMSOL Multiphysics^TM^ (COMSOL 4.3b, COMSOL Inc., Stockholm, Sweden).

## 2. Structure Design and 3D-FEM Simulation

### 2.1. Material and Structure Design of IR Thermal Emitter

There are three factors to consider for the materials used in the hotplate: (1) For high-temperature stability, the materials must be capable of operating at temperature in excess of 1000 °C; (2) For avoiding structure micro-crack due to thermal stress, the mismatch of thermal expansion between materials must be as low as possible; (3) Materials used to fabricate the emitters must be compatible with MEMS processes. The best candidate material should be silicon (Si), and thus a Si-based structure design is considered in our IR thermal emitter.

The designed Si-based MEMS IR emitter consists of a micro-heater, a support layer, and a frame. The micro-heater is fabricated on the support layer over the silicon cavity, forming a structure of hotplate. In order to decrease the power consumption, the hotplate geometry is designed as bridge-style hotplate (BSH) for blocking the heat transfer from heater to frame.

**Figure 1:**
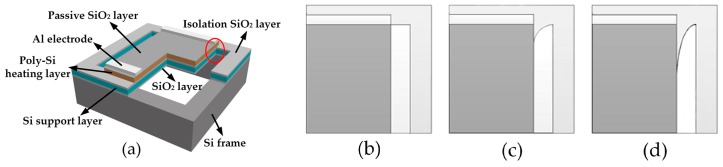
(**a**) Structure design of bridge-style Si-based micro-electromechanical systems (MEMS) infrared (IR) emitter with sectional views in hotplates, and partial enlarged top-views for (**b**) square fillet (SF); (**c**) circular fillet (CF); and (**d**) oval fillet (OF).

As shown in Figure 1a, the BSH hotplate, consisting of a polycrystalline silicon (Poly-Si) heating layer, an electrical isolation SiO_2_ layer, and a self-heating support layer, is constructed in a Si frame. The Poly-Si layer is doped as a resistive layer on which a thin SiO_2_ layer is used to keep Poly-Si from rapid oxidation during high temperature operation. In this design, we need to consider the reliability design in the interior corners between hotplate and frame. It is well known that the stress is generally concentrated in the sharp corner as marked with red circle in Figure 1a. If the concentrated stress exceeds the material’s theoretical cohesive strength, fatigue cracks will generate and further result in structure failure by crack propagation. A method decreasing the stress concentration is to create a smooth blending fillet at the sharp edges of corner. Figure 1b–d show different fillet geometries, respectively, such as the original square fillet (SF), improved circular fillet (CF), and improved oval fillet (OF).

### 2.2. Parameter Setup of 3D-FEM Simulation

Considering the requirements of performance and reliability, the thermal-mechanical stability of the micro-hotplate structure should be considered well, especially the dependences of thermal expansion coefficients on materials. The design can be supported using electro-thermal FEM simulation with software COMSOL Multiphysics^TM^ [14].

The Joule heating and thermal expansion modules are selected in COMSOL^TM^ for the electro-thermal and thermal-stresses simulations. Several material properties are required to solve the mathematical equations embedded in software. Table 1 shows the material properties of polycrystalline silicon (Poly-Si) and single crystal silicon (SC-Si), which were chosen from the build-in Material Library in COMSOL software. Table 2 shows the structure and dimension parameters of emitters used in simulations, which were chosen from reference [13]. The 100-nm thick surface SiO_2_ passive layer and 500-nm thick SiO_2_ electrical isolation layer, which are necessary for the fabrication of emitter, are not considered in this model for simplifying the calculation. In actual device, the mechanical SC-Si support layer is heavily-doped for achieving a self-heating structure with IR absorptivity in excess of 80% in the wavelength range of 2–20 μm [13,15]. However, in this simulation the heavily-doped silicon is assumed to have same material properties with Si except that the electric conductivity is set to zero for the purpose of electrical isolation. Based on the fact that heat transfer equation built-in software is correlated to material parameters, such as density, heat capacity, temperature, and thermal conductivity [14], it is considered that the electrical conductivity of zero has no effect on thermal conduction of heavily-doped silicon layer. Considering the excellent heat transfer system in practical devices, such as packaging socket, metal heat sink, and cooling system, fixed temperature and potentials are set at ends of the heater. Meanwhile, the convection and radiation are also considered at the outer surfaces of the emitter. The surface emissivity of SiO_2_ was set as 0.8 [16]. Room temperature is defined to 20 °C and the applied driving voltage is 12 V. The simulations can be calculated under Dirichlet, Neumann, and mixed boundary conditions numerically in the 3D-FEM model with tetrahedral mesh. The calculations were performed for 40 s with the requirements of 3.8 GB memory.

## 3. Results and Discussion

### 3.1. Electro-Thermal-Mechanical Simulations

**Figure 2:**
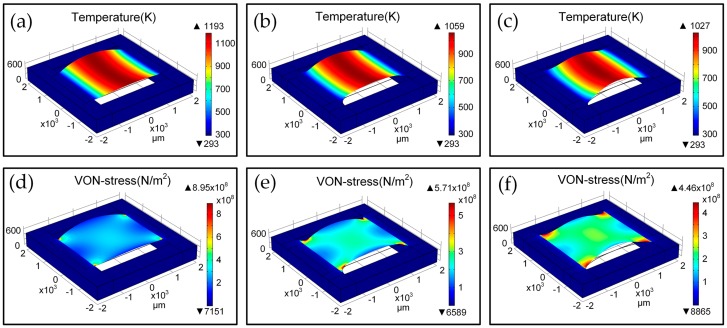
3D thermal-mechanical simulations of bridge-style hotplate (BSH) emitters using software COMSOL^TM^. Thermal distribution and deflection for (**a**) BSH-SF, (**b**) BSH-CF, and (**c**) BSH-OF emitters; Stress distribution and deflection for (**d**) BSH-SF, (**e**) BSH-CF, and (**f**) BSH-OF emitters.

**Figure 3:**
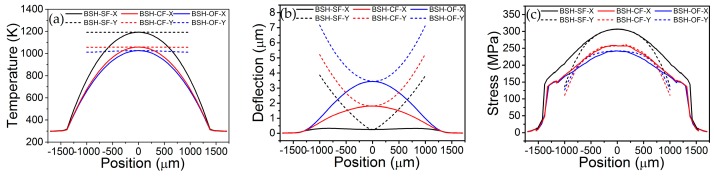
Dependence of temperature (**a**); deflection (**b**); and stress (**c**) on hotplate position along *x* and *y* directions in hotplate center of BSH-SF, BSH-CF, and BSH-OF emitters under work voltage of 12 V.

Figure 2 shows the 3D-FEM simulation results of thermal and stress distributions of BSH emitters with SF (BSH-SF), CF (BSH-CF), and OF (BSH-OF) structure using software COMSOL^TM^. We extract the simulated values of deflection, temperature, and stress along *x* and *y* directions in hotplate center, as shown in Figure 3. With a driving voltage of 12 V, all kinds of hotplates show a symmetrical temperature distribution along *x* and *y* directions. As the thermal conduction pathways are cut off by two opened slots along both sides of bridge-hotplate, the heat could only flow along the bridge to the frame (see Figure 2a–c and Figure 3a). As a result, the temperature values have a gradient variation in *x* direction and are uniform in *y* direction of bridges, which agree well with the experimental results on the temperature distribution in bridge-hotplate structure as referenced in [13]. Moreover, the BSH-CF and BSH-OF structure have larger hotplate area and heat-flux port area in the connection position between bridge and frame than BSH-SF structure, thus BSH-SF structure exhibits higher temperature value than other two structures in same position of hotplate (see Figure 3a). By observing the deflection of hotplate, it is found that all of hotplate profiles show a V-shaped deformation in *y* direction, while in *x* direction of hotplate the BSH-CF and BSH-OF structures show a great arch-shaped deformation instead of the flat surface of BSH-SF structure (see Figure 3b). However, it is suggested that the great deformation do not mean the great stress generated in hotplate. It is seen from Figure 3c that the BSH-SF structure has larger stress than other two hotplates, which indicates that the stresses can be released by structure deformation. Actually, it can be seen that the maximum stresses of hotplates are concentrated on the positions of fillets with 8.9545 × 10^8^ N/m^2^ for SF, 5.7074 × 10^8^ N/m^2^ for CF, and 4.4584 × 10^8^ N/m^2^ for OF, respectively (Figure 2d–f). Obviously, the maximum stress in OF is reduced by half in comparison with the SF, but the distribution area of stress is increased greatly.

**Figure 4:**
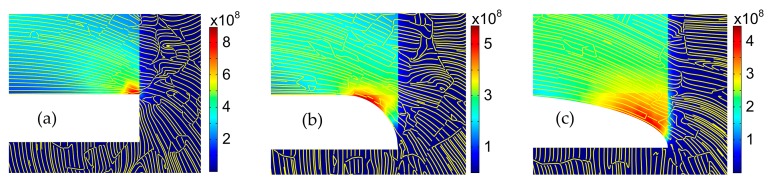
Distributions of stresses and stresses streamlines in SF (**a**); CF (**b**); and OF (**c**) structures.

Stress streamlines, which are a family of curves that are instantaneously tangent to the force vector of the stress flow, show the direction of stress variation at any point. Figure 4 exhibits the distributions of simulated stresses and stress streamlines in SF, CF, and OF structure. It can be seen from Figure 4c that the OF structure gives the smoother flows of stress streamlines than SF and CF structures. The stress flows in OF structure have a smooth and slow transition from the low stress area to the high stress area, resulting in the decrease of stress concentration.

### 3.2. Simulations of IR Emission Spectra

**Figure 5:**
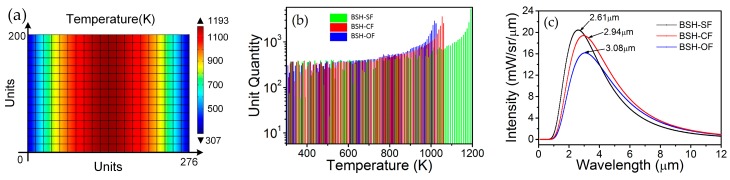
(**a**) Rectangle mesh in the heater of BSH-SF emitter; (**b**) Statistical comparison of temperature distributions of BSH-SF, BSH-CF, and BSH-OF emitters at driving voltage of 12 V; (**c**) Calculated IR spectra for theBSH-SF, BSH-CF, and BSH-OF emitters at driving voltage of 12 V.

The radiation characteristics of IR thermal emitters can be investigated by calculating the IR emission spectra of emitters, which are related to surface temperature and surface emissivity of emitters. In this simulation, the surface emissivity coefficient of the SiO_2_ passivation layer is set as 0.8. According to the Planck’s radiation law, the spectral radiance *E* can be calculated using the following equation:

(1) $$E(\lambda ,T)=\varepsilon \cdot \frac{2hc^{2}}{\lambda^{5}}\left(\frac{1}{e^{\frac{hc}{k_{B}\lambda T}-1}}\right)$$

where ε the emissivity coefficient, *k_B_* the Boltzmann constant, *h* the Planck constant, and *c* the speed of light in the medium. For extracting the values of temperatures distributed in heater surface, a statistical approach is used to quantify the thermal distribution. The heater areas are divided equally into 276 × 200 units for BSH-SF, BSH-CF, and BSH-OF structure, and then the temperature value is extracted from each unit, as shown in Figure 5a.

Figure 5b shows a statistical comparison of temperature distribution of three kinds of hotplate structures at driving voltage of 12 V. By calculating the ratios between the amount of units of temperature larger than 700 K and the total amount of units, we found that the ratios are 76.8%, 69.3% and 66.6% for BSH-SF, BSH-CF, and BSH-OF structures, respectively. The statistical results indicate that the BSH-SF structure has a wider temperature range and a larger area of high temperature region, and thus a higher lighting intensity and electro-optical conversion efficiency than other two kinds of structure. According to the extracted temperature value in each unit, we calculated the IR spectra of all units and combined them into an ultimate spectrum for each kind of structures using Equation (1). As shown in Figure 5c, the wavelengths in peak positions are 2.61 μm, 2.94 μm and 3.08 μm for BSH-SF, BSH-CF, and BSH-OF structures, respectively. According to the Wien displacement law, the equivalent temperature *T* can be calculated using follow equation:

(2) *T* = Λ/λ_max_

here, Λ is the Wien’s displacement constant, equal to 2.897 × 10^−3^ m·K, and λ_max_ is the wavelength in peak. The equivalent temperatures are 1110, 985, and 941 K for BSH-SF, BSH-CF, and BSH-OF structures, respectively. While the BSH-SF emitter has higher emission intensity than other two emitters, considering the structure reliability, the BSH-OF is still best candidate. We also found that the peak-value wavelength of BSH-SF in simulation (2.61 μm) is less than that in experiment (2.97 μm [13]), which implies that the simulated equivalent temperature of BSH-SF is 135 K higher than that experimental one. This can be attributed to the packaging system of actual devices, which was not considered in simulation.

### 3.3. Simulation of Modulation Characteristics

**Figure 6:**
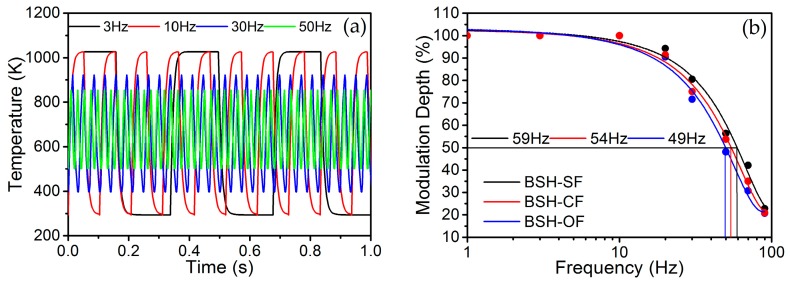
(**a**) Temperature response characteristics of BSH-OF emitter for typical 3, 10, 30, and 50 Hz of square wave voltages; (**b**) Comparison of frequency responses of BSH-SF, BSH-CF, and BSH-OF emitters.

The basic modulation principle of IR emitter is that of pulsed electrical heating followed by rapid radiation self-cooling [16]. The modulation characteristics of IR emitters can be characterized using the dependences of modulation depth on frequency. The modulation depth *m*(*f*) of IR emitter can be calculated by following equation:

(3) $$m\left(f\right)=\frac{T{\left(f\right)}_{\text{p-p}}}{T{\left(1\;\text{Hz}\right)}_{\text{p-p}}}\times 100\%$$

here, *T*(*f*)_p-p_ is the peak-to-peak value of response temperature of IR emitter driven by a square wave voltage with frequency *f*. The dependence of temperature on time can be achieved using the transient solution model in electro-thermal FEM simulation. Figure 6a shows the temperature response characteristics of BSH-OF emitter for typical 3, 10, 30 and 50 Hz of square wave voltages, respectively.

It is found that the peak-to-peak value of response temperature decreases with increasing the modulation frequency. By observing the voltage waveform of 3 Hz signal, the warm-up time is 22 ms and the decay time 15 ms. According to the peak-to-peak values of different response temperature curves, the modulation depth of IR emitter can be calculated using Equation (3). Figure 6b shows a comparison of frequency responses of BSH-SF, BSH-CF, and BSH-OF structures. The −3.0 dB bandwidths of frequency response of BSH-SF, BSH-CF, and BSH-OF structures, corresponding to a frequency range for modulation depth from 1 to 1/$\sqrt{2}$, are 59 Hz, 54 Hz and 49 Hz, respectively. The effect of hotplate on modulation frequency should be attributed to the thermal-mass variation of fillet. Generally, the actual −3.0 dB bandwidth of emitter should be lower than simulated value when the package and driving voltage are considered.

## 4. Conclusions

In summary, we designed and simulated silicon-based MEMS IR thermal emitters for gas sensing application. The IR thermal emitter is designed as a BSH structure suspended on a silicon frame for realizing a good thermal isolation between hotplate and frame. Three kinds of fillet structures, namely square fillet, circular fillet and oval fillet, are considered to use in the contact corner between hotplate and frame for increasing the reliability of BSH structure in high temperature. A 3D-FEM using software COMSOL^TM^ is used to analyze the electro-thermal, thermal-mechanical, and thermal-optical properties of BSH IR emitter. The simulation results show that the BSH with oval fillet has the lowest stress distribution and smoothest flows of stress streamlines, while the BSH with square fillet has the highest temperature and stress distribution. The thermal-optical and thermal-response simulations further indicate that the BSH with oval fillet is an optimal design for a reliable IR thermal emitter in spite of having slight inadequacies in emission intensity and modulation bandwidth in comparison with the other two structures.

## Figures and Tables

**Table 1:** Material properties used in COMSOL Multiphysics^TM^.

Parameters	Single Crystal Silicon (SC-Si)	Polycrystalline Silicon (Poly-Si)
Density	2329 kg/m^3^	2320 kg/m^3^
Young’s modulus	170 × 10^9^ Pa	160 × 10^9^ Pa
Poission’s ratio	0.28	0.22
Thermal expansion coefficient	2.6 × 10^−6^ K^−1^	2.6× 10^−6^ K^−1^
Heat capacity	700 J/(kg·K)	678 J/(kg·K)
Thermal conductivity	131 W/(m·K)	34 W/(m·K)

**Table 2:** Structure and dimension parameters of emitters used in simulations.

Parameters	Length (μm)	Width (μm)	Thickness (μm)
Frame	4000	4000	400
Cavity	2760	2760	400
Hotplate	3400	2000	5.5
Slot	2760	380	5.0
Electrode	2000	200	5.0
Fillet	Circular: *R* = 380 μm		5.0
	Oval: *a* = 1380 μm, *b* = 380 μm		
